# Optimal Homeostasis Model Assessment of Insulin Resistance (HOMA-IR) Cut-Offs: A Cross-Sectional Study in the Czech Population

**DOI:** 10.3390/medicina55050158

**Published:** 2019-05-17

**Authors:** Dagmar Horáková, Ladislav Štěpánek, Vladimír Janout, Jana Janoutová, Dalibor Pastucha, Helena Kollárová, Alena Petráková, Lubomír Štěpánek, Roman Husár, Karel Martiník

**Affiliations:** 1 Department of Public Health, Faculty of Medicine and Dentistry, Palacký University Olomouc, Hněvotínská 3, 77515 Olomouc, Czech Republic; dagmar.horakova@upol.cz (D.H.); janout@lf.upol.cz (V.J.); helena.kollarova@upol.cz (H.K.); alena.petrakova@upol.cz (A.P.); roman.husar@upol.cz (R.H.); 2 Department of Healthcare Management, Faculty of Health Sciences, Palacký University Olomouc, Hněvotínská 3, 77515 Olomouc, Czech Republic; jana.janoutova01@upol.cz; 3 ReFit Clinic s.r.o., U Reálky 4, 77900 Olomouc, Czech Republic; dalibor.pastucha@refitclinic.cz; 4 Institute of Biophysics and Informatics, First Faculty of Medicine, Charles University, Salmovská 1, 12000 Prague, Czech Republic; Lubomir.Stepanek@lf1.cuni.cz; 5 Ambulance Prof. MUDr. Karla Martiníka DrSc. s.r.o, Bratří Štefanů 895, 50003 Hradec Králové, Czech Republic; ambulance@profmartinik.cz

**Keywords:** insulin resistance, type 2 diabetes mellitus, prediabetes, HOMA-IR, cut-off point

## Abstract

*Background and Objectives:* The key pathogenetic mechanism of glucose metabolism disorders, insulin resistance (IR), can be assessed using the Homeostasis Model Assessment of IR (HOMA-IR). However, its application in clinical practice is limited due to the absence of cut-offs. In this study, we aimed to define the cut-offs for the Czech population. *Methods:* After undergoing anthropometric and biochemical studies, the sample of 3539 individuals was divided into either nondiabetics, including both subjects with normal glucose tolerance (NGT, *n* = 1947) and prediabetics (*n* = 1459), or diabetics (*n* = 133). The optimal HOMA-IR cut-offs between subgroups were determined to maximize the sum of the sensitivity and specificity for diagnosing type 2 diabetes mellitus (T2DM) or prediabetes. The predictive accuracy was illustrated using receiver operating characteristic (ROC) curves. Logistic regression was performed to assess the association between a target variable (presence/absence of T2DM) depending on the HOMA-IR score as well as on the age and sex. *Results:* The HOMA-IR cut-off between nondiabetics and diabetics for both sexes together was 3.63, with a sensitivity of 0.56 and a specificity of 0.86. The area under the ROC curve was 0.73 for T2DM diagnosing in both sexes. The HOMA-IR cut-off between the NGT subjects and prediabetics was 1.82, with a sensitivity of 0.60 and a specificity of 0.66. Logistic regression showed that increased HOMA-IR is a risk factor for the presence of T2DM (odds ratio (OR) 1.2, 95% confidence interval (CI) 1.14–1.28, *p* < 0.0001). The predictive ability of HOMA-IR in diagnosing T2DM is statistically significantly lower in females (OR 0.66, 95% CI 0.44–0.98). The results are valid for middle-aged European adults. *Conclusions:* The results suggest the existence of HOMA-IR cut-offs signaling established IR. Introduction of the instrument into common clinical practice, together with the known cut-offs, may contribute to preventing T2DM.

## 1. Introduction

The global burden of type 2 diabetes mellitus (T2DM) is expected to double by 2030, with impacts on both the health systems and economies of affected countries. There are growing numbers of diabetics not only in affluent countries but also in low- and middle-income countries [1]. T2DM has a long prodromal or preclinical period with fasting as well as postprandial blood glucose levels ranging from normal to pathological. This period is referred to as prediabetes. For its clinical significance, prediabetes has recently been considered a distinct nosological entity. Prediabetes is an asymptomatic condition that has to be actively screened for during preventive or follow-up examinations. Prediabetes increases the risk of atherosclerosis or certain cancers and predicts the development of T2DM [2]. In individuals with prediabetes, both basic etiopathogenetic conditions typically seen in T2DM patients are present, namely, the insulin resistance (IR) and insulin deficiency (impaired B cell secretion). IR syndrome is one of the important public health issues. For simple screening, efficient tools have been sought. One of them is the Homeostasis Model Assessment of IR (HOMA-IR). Its use in routine clinical practice is limited due to an absence of accurate thresholds, with only some countries recommending cut-off points for their populations [3,4,5]. 

T2DM is a disease state with distortions in the relationship between circulating glucose and insulin values in a manner that does not reflect the systemic insulin sensitivity. Hyperglycemia is the hallmark of T2DM and is accompanied by “glucose toxicity” with respect to insulin secretion. Consequently, studies assessing the relationship between indices based on fasting plasma glucose (FPG) and insulin levels and clamp measures could reflect falsely inflated slopes and correlation coefficients in regression equations when data from nondiabetic and diabetic subjects are included in the same regression analyses [6]. That is why our work separately distinguishes subjects with T2DM, prediabetes and normal glucose tolerance.

The present study defines HOMA-IR cut-offs for middle-aged male and female in the Czech Republic.

## 2. Materials and Methods

### 2.1. Patients and Samples

The sample comprised 3539 middle-aged individuals examined in the co-author’s internal medicine center in Hradec Králové who agreed to participate. Data were collected in 2009–2015. The sample was divided into three subgroups: subjects with normal glucose tolerance (NGT, *n* = 1947), prediabetics (*n* = 1459) and diabetics (*n* = 133). The NGT subjects together with the prediabetics formed a subgroup of nondiabetics, which was used for the HOMA-IR cut-off analysis. The prediabetics were individuals with at least one of the following conditions: (1) FPG 5.6–6.9 mmol/L; (2) 120-minute plasma glucose 7.8–11.0 mmol/L after a 75 g oral glucose tolerance test (OGTT). The diabetics were exclusively individuals with T2DM, which was defined as (1) FPG ≥ 7.0 mmol/L; or (2) 120-minute plasma glucose ≥11.1 mmol/L after OGTT. Most of the diabetics received long-term therapy with oral antidiabetic drugs, most frequently metformin. Insulin therapy was an exclusion criterion as well as very high FPG which would limit the use of the HOMA-IR. Individuals in the NGT subgroup included the other patients attending the center with normal glucose metabolism.

All procedures were in accordance with institutional and national ethical standards. The need for approval was waived by the Ethics Committee of University Hospital and Faculty of Medicine and Dentistry of the Palacký University. For being included in the study, all subjects signed informed consent forms after they were explained all information regarding the project.

### 2.2. Laboratory Analysis 

Venous blood samples were drawn in the morning after a 12-hour fast. After centrifugation, the serum was used for analyses on the day of blood collection. Routine serum biochemical parameters were analyzed on COBAS 8000 (Roche Diagnostics GmbH, Manheim, Germany). Insulin was determined by the Chemiluminescent Microparticle Immunoassay method on Architect i1000SR (Abbott Laboratories, Chicago, IL, USA). All analyses were performed according to the manufacturer’s instructions and after verification of methods.

### 2.3. Statistical Analysis 

The HOMA-IR was calculated with the following formula (glucose levels in mmol/L, insulin levels in mIU/L) [7]:

$$HOMA-IR=\;\frac{glucose\times insulin}{22.5}$$

The Wilcoxon signed-rank test (p-value) was used to compare numerical characteristics between the given subgroups. For each sex, cut-offs were determined so that the sum of sensitivity and specificity was maximized. Sensitivity and specificity were considered equally important and, therefore, their relative weights in the sum were 1:1. In other words, we searched for the maximum of Youden’s index as a function of sensitivity and specificity [8]. The accuracy and predictive ability of the cutoffs are illustrated using receiver operating characteristic (ROC) curves for both sexes together and separately. A logistic regression with a logit link function was used to assess the association between the logarithm of the odds to diagnose T2DM depending on the HOMA-IR score. The level of statistical significance was set at *p* < 0.05. A confusion matrix comparing the actual presence of the diagnosis of T2DM against its prediction was performed. A statistical analysis was performed using the computing environment R (R Foundation for Statistical Computing, Vienna, Austria; http://www.r-project.org/). 

## 3. Results

### 3.1. Characteristics of the Study Population 

The basic characteristics of all subjects in the three subgroups are shown in Table 1. The NGT subgroup consisted of 1590 females and 357 males with a mean age of 38.5 years. Their metabolic parameters (glucose and insulin levels; total, HDL and LDL cholesterol, triglycerides) were normal; their BMI was in the obesity range. The prediabetic subgroup included 1047 females and 412 males with a mean age of 44 years. Their metabolic parameters were statistically significantly worse than in the NGT subgroup. The diabetic subgroup comprised 82 females and 51 males with a mean age of 53.6 years. Their blood glucose levels, both fasting and after a glucose load, were typical for diabetics; their BMI was also in the obesity range. The last line of Table 1 demonstrates significantly growing mean values of the HOMA-IR from the NGT subjects to diabetics.

### 3.2. HOMA-IR Cut-Offs

**Figure 1:**
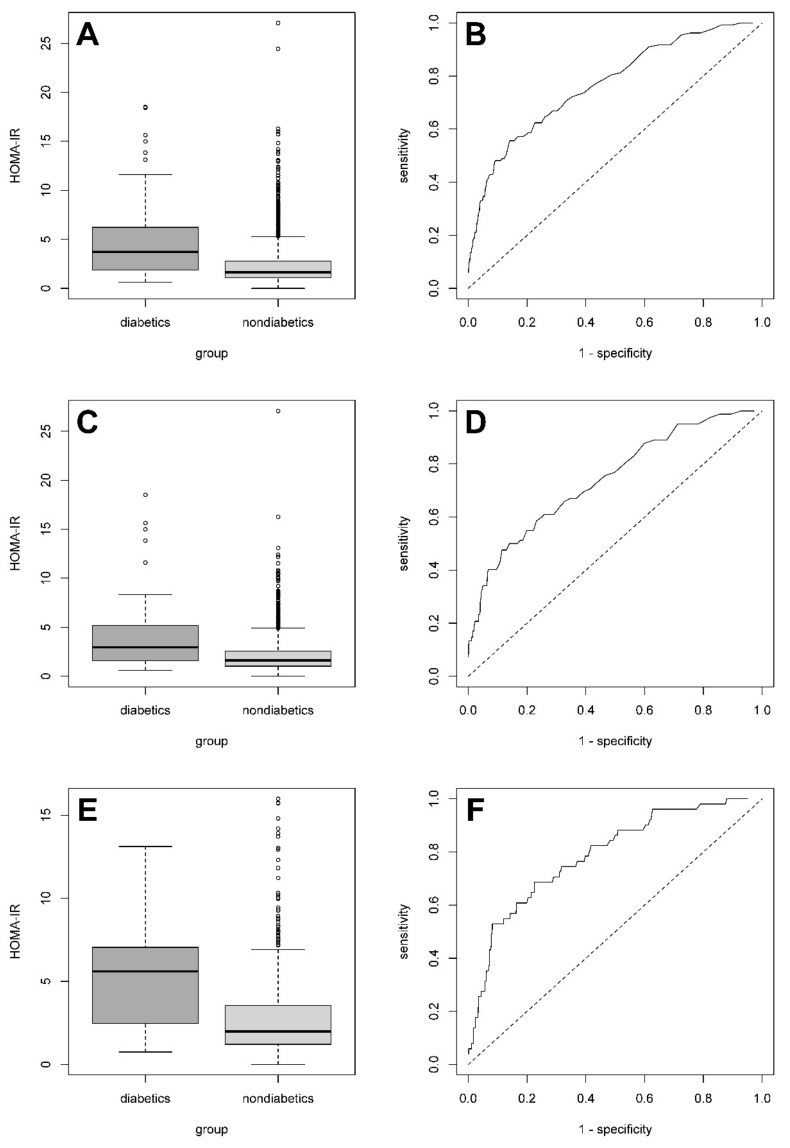
The boxplots depicting an association between Homeostasis Model Assessment of Insulin Resistance (HOMA-IR) scores and type 2 diabetes mellitus (T2DM) diagnosis in both sexes (**A**), females (**C**) and males (**E**). Receiver operating characteristic curves for T2DM prediction using cut-offs of the HOMA-IR scores in both sexes (**B**), females (**D**) and males (**F**).

Table 2 shows HOMA-IR cut-offs maximizing the sum of the sensitivity and specificity for T2DM in the sample. The HOMA-IR cut-off separating nondiabetics (NGT and prediabetics) and diabetics based on their IR in the entire sample, regardless of sex, was 3.63. If a patient’s HOMA-IR score is greater than 3.63, then regardless of his/her sex, he/she has a significantly higher chance of having T2DM. The same value, with a slightly smaller sum of sensitivity and specificity, was calculated for females alone. In males, the HOMA-IR cut-off was 3.69; they had the highest sum of sensitivity and specificity. Figure 1A,C,E show, in the form of boxplots, the association between HOMA-IR scores and T2DM diagnosis in both sexes, females and males, respectively. The similarity of the characteristics, including box shifts in the boxplots, suggests the existence of a constant relationship between HOMA-IR scores and the presence of T2DM. Figure 1B,D,F show ROC curves for HOMA-IR scores in both sexes, females and males, respectively. The adequate upper half-plane convexity of the curves suggests that the predictive ability of HOMA-IR in diagnosing T2DM is not negligible. The area under the ROC curve was 0.7291 (95% confidence interval (CI) 0.7272-0.7310) for T2DM diagnosing using the cut-offs in both sexes, 0.7075 (95% CI 0.7055–0.7096) in females, and 0.7382 (95% CI 0.7381–0.7383) in males. The HOMA-IR cut-off distinguishing subjects with NGT and prediabetics was 1.82 (sensitivity 0.60, specificity 0.66). The HOMA-IR cut-off separating prediabetics and diabetics had the same value as in case of all nondiabetics and diabetics (3.63) with very similar sensitivity (0.56) and slightly lower specificity (0.78).

### 3.3. Logistic Regression

Table 3 shows odds ratios (ORs) and their 95% CI for coefficient estimates from various regression models. HOMA-IR scores statistically significantly influence the odds that T2DM is diagnosed; they increase the odds by 1.2043-fold per each unit of HOMA-IR enlargement (*p* < 0.0001). For HOMA-IR, a statistically significant predictor is sex; when HOMA-IR scores are used to estimate the odds of T2DM, with the other variables remaining unchanged, the odds of T2DM are 0.6567-fold higher in females compared to males (*p* = 0.0384). Another statistically significant predictor is age, but with OR close to one (*p* < 0.0001). The accuracy of the prediction for the logistic regression model with the HOMA-IR predictor is 0.962 to 0.963 (*p* < 0.0001) according to the confusion matrix.

## 4. Discussion

The results suggest the existence of HOMA-IR cut-offs signaling established IR. This condition is one of the main pathogenetic mechanisms of T2DM, atherosclerosis and metabolic syndrome which increasingly contribute to the morbidity and mortality of the affluent populations [9]. The main clinical manifestation of increasing IR is dysglycemia. To assess IR, a number of methods have been introduced. The hyperinsulinemic euglycemic glucose clamp, albeit the gold standard technique, is limited by its complexity, invasiveness, high cost, and laborious requirements. HOMA-IR, on the contrary, provides a convenient and inexpensive means of estimating IR and the derived estimates have been shown to correlate very well with those derived from the euglycemic clamp [10,11]. The evaluation of IR is useful in clinical practice. Therefore, the assessment of IR via HOMA-IR is a key index for the primary prevention of T2DM and is thus found in guidelines for the screening of high-risk groups [4]. To distinguish healthy individuals from those with IR, knowing HOMA-IR cut-offs is essential. However, the value has not been clearly defined for many European populations. Therefore, the present study aimed to determine the HOMA-IR cut-off for the Czech population. The results are very similar to those reported in three European countries (Sweden 2.0, France 3.8 and Portugal 2.33) [4]. In a cross-sectional study of middle-aged Chinese people, Lee et al. found that a HOMA-IR cut-off of 1.97 was able to discriminate nondiabetics from diabetics [5]. A clear definition is limited by the interpretation of blood glucose levels itself as these change considerably over time [12]. Moreover, there are both ethnic differences in cut-offs and gender variations [3,13]. The latter was observed in the present study, with the cut-off for females being slightly lower than that for males. The accuracy of prediction for the logistic regression model with the HOMA-IR predictor in our analysis is 0.962 to 0.963 (*p* < 0.0001) according to the confusion matrix.

One of the factors potentially influencing insulin sensitivity is the increased resting metabolic rate, as shown in a large cohort of 782 adults [14]. It has also been reported that insulin sensitivity may be affected by the total lean body mass as the target organ for insulin activity, with the percentage of total body mass that is lean being typically higher in males [15]. Even though individuals in the present study were obese, given their normal glucose (even after a glucose load) and lipid metabolism, their obesity may be considered as metabolically healthy that may not considerably affect the results [16]. Subsequently, with the primary accumulation of adipose tissue in the organism, these individuals may be expected to develop IR [17].

IR and associated metabolic abnormalities are increasingly being reported in the adolescent population. The HOMA-IR cut-off as an indicator of metabolic syndrome in adolescents has not been established. In Indian adolescents, HOMA-IR scores increased with sexual maturity and with progression from normal to obese. A HOMA-IR cut-off of 2.5 provided the maximum sensitivity and specificity in diagnosing [18]. Obesity in children and adolescents is associated with higher HOMA-IR scores. In boys with obesity, IR increased at the end of pubertal maturation, with a delay in puberty. These findings should be taken into account when establishing HOMA-IR cut-offs for the pubescent population [19]. Therefore, the use and interpretation of HOMA-IR scores in adolescents should be considered together with their state of pubertal maturation.

HOMA-IR is a robust instrument currently used mostly in large population studies to identify IR already present in prediabetes. Prediabetes is a high-risk state for T2DM, especially in patients who remain with prediabetes despite intensive lifestyle intervention. Reversion to normal glucose regulation, even if transient, is associated with a significantly reduced risk of future T2DM [20]. It is, therefore, essential to develop methods for the early detection of prediabetes, apply them in practice and then use targeted intervention to reduce the risk for developing T2DM. In addition to standard methods based on measuring blood glucose levels (both fasting and after a glucose load) and glycated hemoglobin, HOMA-IR may be potentially used. However, to apply HOMA-IR in common practice, it is necessary to know the cut-off signaling established IR together with factors that may influence the cut-off. What is more, the cut-off values of HOMA-IR need to be examined in subjects with NGT in order to devise a standard for the primary prevention of prediabetes and T2DM [4]. This is a major challenge for further research. The sensitivity of HOMA-IR detection must also be studied. However, with regard to the sensitivity of the reached HOMA-IR cut-offs, it cannot be recommended as a reliable screening test. The reported positive predictive value is low due to the fact that the overall prevalence of T2DM in the sample is relatively low while the reported negative predictive value is high enough in females, males and the whole sample, which supports the good specificity of the cut-offs.

### Limitations of the Study

The study has its limitations. In all subjects, the HOMA-IR scores were calculated based on the single measurements of blood glucose and insulin levels at a single time point; these may change over a short period of time. The diabetic subgroup for identification of the cut-off is much smaller than without the diabetes subgroup. The euglycemic clamp to confirm the presence of IR was not performed. The validity of the HOMA-IR should be carefully considered in subjects with a lower BMI, lower beta cell function, and high FPG such as in T2DM with the insulin secretory defect [21]. However, all subjects in the study had a BMI in the obesity range and subjects with very high fasting glucose levels were excluded. Individuals in the diabetic group mostly used oral antidiabetic drugs which may have affected their HOMA-IR scores. 

## 5. Conclusions

Given the current global epidemic of obesity, it is rather desirable to prevent T2DM. Using data on a group of individuals collected over a period of six years, the HOMA-IR cut-off of 3.63 was determined for the middle-aged Czech population. These may identify patients with already established IR at the stage of T2DM. However, the sensitivity of the cut-offs was 0.56. Therefore, this test cannot currently be recommended as a reliable tool for IR screening. The specificity of the test (0.86) confirms its good validity. The HOMA-IR cut-off between subjects with NGT and prediabetics was 1.82 (sensitivity 0.60, specificity 0.66), and thus prediabetes is bordered with the HOMA-IR values of 1.82 and 3.63. The determination of the optimal cut-off for diagnosing at-risk individuals in common practice is beneficial for early detection of prediabetes.

## Figures and Tables

**Table 1:** The basic metabolic and clinical characteristics of subjects.

Characteristics	NGT	*p*-Value NGT vs. Prediabetics	Prediabetics	*p*-Value Prediabetics vs. Diabetics	Diabetics
N	1947 (F1590, M357)		1459 (F1047, M412)		133 (F82, M51)
Age (years)	38.5 (38.0; 39.1)	<0.0001	44.0 (43.4; 44.6)	<0.0001	53.6 (51.8; 55.4)
Glucose (mmol/L)	5.15 (5.11; 5.18)	<0.0001	5.82 (5.79; 5.84)	<0.0001	6.59 (6.30; 6.88)
Insulin (mIU/L)	7.93 (7.67; 8.19)	<0.0001	10.73 (10.12; 11.34)	<0.0001	15.32 (13.27; 17.36)
Total cholesterol (mmol/L)	5.01 (4.97; 5.05)	<0.0001	5.25 (5.20; 5.30)	0.0111	5.01 (4.84; 5.19)
HDL cholesterol (mmol/L)	1.51 (1.50; 1.53)	<0.0001	1.38 (1.36; 1.40)	0.0222	1.31 (1.24; 1.38)
LDL cholesterol (mmol/L)	2.94 (2.90; 2.97)	<0.0001	3.14 (3.10; 3.19)	0.1984	3.01 (2.86; 3.16)
Triglycerides (mmol/L)	1.26 (1.21; 1.31)	<0.0001	1.54 (1.48; 1.61)	0.3554	1.63 (1.38; 1.87)
BMI (kg/m^2^)	31.60 (31.26; 31.94)	<0.0001	35.04 (34.65; 35.42)	<0.0001	37.81 (36.39; 39.23)
HOMA-IR	1.47 (1.43; 1.51)	<0.0001	2.17 (2.10; 2.25)	<0.0001	3.49 (3.04; 3.99)

NGT, normal glucose tolerance; F, female; M, male; HOMA-IR, homeostasis model assessment of insulin resistance.

**Table 2:** The Homeostasis Model Assessment of Insulin Resistance (HOMA-IR) cut-offs maximizing the sum of sensitivity and specificity for the diagnosis of type 2 diabetes mellitus (T2DM).

Sample	HOMA-IR Cut-Off	Sensitivity	Specificity	Positive PV	Negative PV	Accuracy
Females	3.634	0.476	0.885	0.094	0.979	0.873
Males	3.691	0.686	0.775	0.142	0.971	0.770
Females + males	3.634	0.556	0.859	0.112	0.978	0.848

HOMA-IR, homeostasis model assessment of insulin resistance; T2DM, type 2 diabetes mellitus; PV, predictive value.

**Table 3:** The odds ratios (ORs) of linear coefficients and their 95% CIs of multivariate logistic regression with the presence of T2DM as a dependent variable.

Predictors	OR	2.5% Quantile of OR	97.5% Quantile of OR	*p*-Value
(intercept)	0.0007	0.0003	0.0018	<0.0001
HOMA-IR	1.2043	1.1365	1.2759	<0.0001
Sex = female	0.6567	0.4431	0.9841	0.0384
Age	1.0824	1.0656	1.1001	<0.0001

OR, odds ratio; CI, confidence interval; HOMA-IR, homeostasis model assessment of insulin resistance; T2DM, type 2 diabetes mellitus.
